# Gut microbiota metabolism of branched‐chain amino acids and their metabolites can improve the physiological function of aging mice

**DOI:** 10.1111/acel.14434

**Published:** 2024-12-04

**Authors:** Hongchao Wang, Ling Feng, Zhangming Pei, Jianxin Zhao, Shourong Lu, Wenwei Lu

**Affiliations:** ^1^ State Key Laboratory of Food Science and Technology Jiangnan University Wuxi Jiangsu China; ^2^ School of Food Science and Technology Jiangnan University Wuxi Jiangsu China; ^3^ The Affiliated Wuxi People's Hospital of Nanjing Medical University, Wuxi People's Hospital, Wuxi Medical Center Nanjing Medical University Wuxi Jiangsu China; ^4^ National Engineering Research Center for Functional Food Jiangnan University Wuxi Jiangsu China

**Keywords:** aging, branched‐chain amino acids, gut microbiota, metabolism, mice

## Abstract

The metabolism of branched‐chain amino acids by gut microbiota can improve overall health and may reverse aging. In this study, we investigated *Parabacteroides merdae,* a gut microbe that is known to catabolise branched‐chain amino acids (BCAAs). Three metabolites of BCAAs isovalerate, 2‐methylbutyrate, and isobutyrate were used to treat D‐gal induced aging mice. The results showed that these treatments could delay aging in mice by providing health benefits in reducing oxidative stress and inflammation, improving muscle capacity, reversing brain acetylcholine levels, and regulating blood glucose. The mechanism was preliminarily explored by combining the gut microbiota metagenome and faecal serum metabolome. *Parabacteroides merdae* altered the species composition and structure of the gut microbiota in mice. Increasing the abundance of beneficial bacteria, such as *Bifidobacterium pseudolongum*. Three metabolites affects the gut microbiota and the body's pathways of protein and improves the overall health through a variety of signaling pathways. Overall, regulating the gut microbiota involved in branched‐chain amino acid metabolism to bring health benefits may be a new way of reversing aging.

## Graphical abstract

*Parabacteroides merdae* in gut microbiota metabolizes branched‐chain amino acids into isovalerate, 2‐methylbutyrate, and isobutyrate, with key gene porA being less abundant in unhealthy elderly. *Parabacteroides merdae* and its three BSCFAs improve physiological functions in aging mice, with preliminary mechanism exploration through microbiome metagenomics and serum metabolomics.

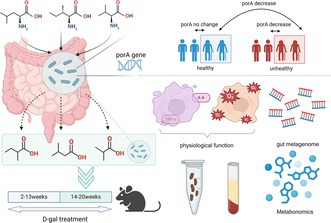

**Abbreviations**
2‐MB: 2‐methylbutyrate
AChE: acetylcholinesterase
ACVD: acute cardiovascular disease
AMPK: AMP‐activated protein kinase
BCAAs: branched‐chain amino acids
BCAT: branched‐chain amino acidtransaminase
BCKA: branched‐chain side‐keto acids
BCKDH: branched‐chain keto aciddehydrogenase
BSCFAs: branched‐chain short‐chain fattyacids
CAT: catalase
CRC: colorectal cancer
CVD: cardiovascular diseases
FI: frailty index
GSH‐px: glutathione peroxidase
IL‐6: interleukin‐6
ISB: isobutyrate
ISV: isovalerate
MDA: malondialdehyde
mTOR: mammalian target of rapamycin
NNDs: neurodegenerative diseases
OGTT: glucose tolerance curve analysis
PCA: principal component analysis
SCFAs: short chain fatty acids
SOD: superoxide dismutase
T2D: Type 2 diabetes
TCA cycle: citrate cycle
TNF‐alpha: tumor necrosis factor α

## 1 INTRODUCTION

As people live longer worldwide, the aging population grows, making healthy aging a pressing issue. Dysbiosis is a key feature of aging (Lopez‐Otin et al., 2022), influenced by factors such as age‐related changes, diet, environment, and lifestyle habits. The types of gut microbiota and the substances they produce in the gut help maintain normal bodily functions and are important for promoting healthy aging. recently, an increasing number of studies have focused on harnessing gut bacteria to support healthy aging (Milenkovic et al., 2023).

Branched‐chain amino acids (BCAAs), including isoleucine, leucine, and valine, are three essential amino acids in the human body that cannot be synthesised by the body itself and must be obtained from the diet. Recent research has shown that BCAAs are vital in managing metabolic well‐being and the aging process through their influence on signaling pathways, such as the mammalian target of rapamycin (mTOR) and AMP‐activated protein kinase (AMPK) pathways. Branched chain amino acid intake in diet (Richardson et al., 2021; Solon‐Biet et al., 2019; Yu et al., 2021) may affect the health and metabolism in mice. BCAAs, especially isoleucine restriction, can activate certain nerve‐promoting hunger, which can be induced by starvation, allowing increased food intake during life extension (Weaver et al., 2023). In addition, BCAAs levels are closely associated with metabolic health. The metabolism of the three BCAAs in the body is essential for metabolic health and a healthy lifespan.

In our previous study, artificial intelligence techniques have been employed to develop models for predicting age, based on gut microbiota composition. In addition to assessing individual aging health status, the model interpretation suggests that the metabolism of BCAAs is significantly correlated with age changes. Specifically, the degradation pathways of leucine and isoleucine have been identified as key markers of gut microbiota biological age (Wang et al., 2024). The metabolism of BCAAs by gut microbiota may serve as a crucial factor influencing the host aging process.

**FIGURE 1:**
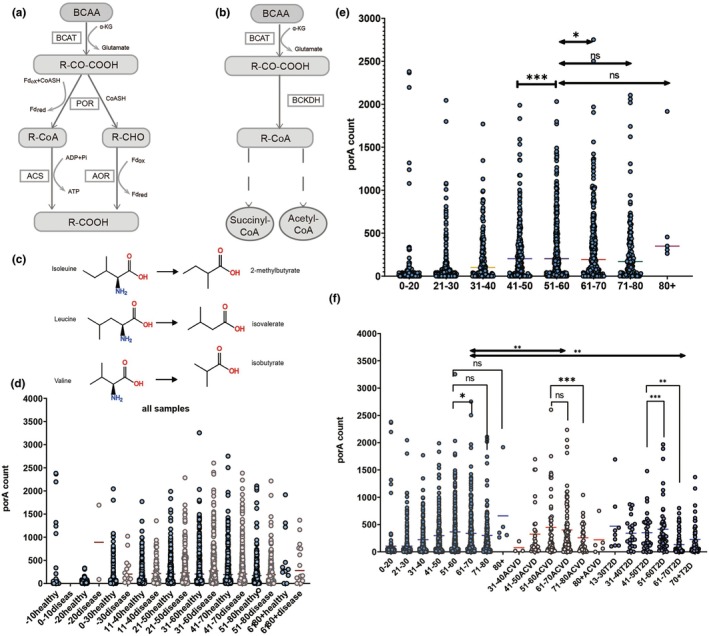
Metabolic pathways of branched‐chain amino acids and metabolites of gut microbiota and Distribution and abundance of porA gene in the human gut microbiomes. (a, b) The gut microbe *Parabacteroides merdae, mediated by* porA genes, metabolised leucine, isoleucine and valine into isovalerate, 2‐methylbutyrate, and isobutyrate, respectively. (c) Chemical structure formula of branched‐chain amino acids and their gut microbiomes metabolites. (d) porA gene in the whole population sample distribution. (e) distribution and abundance of porA genes in healthy individuals. (f) Differences in distribution and abundance of porA genes in healthy individuals and those with ACVD and T2D. *p < 0.05, **p < 0.01, and ***p < 0.001. Data shown are mean ± s.e.m. Statistical analysis was performed using T test.

Gut microbiota is an important regulator of branched‐chain amino acid metabolism. In many human tissues, such as muscle, liver, and brown fat, branched‐chain amino acid transaminase (BCAT) metabolises BCAAs to branched‐chain side‐keto acids (BCKA), and branched‐chain keto acid dehydrogenase (BCKDH) oxidises BCKA. Acetyl‐CoA and succinyl‐CoA are eventually produced and enter the tricarboxylic acid cycle (Lei et al., 2020; Neinast et al., 2019). This is a classical pathway of amino acid metabolism, whereas the branched‐chain amino acid metabolism pathway in intestinal microorganisms is atypical and unique. One study reported that porA gene mediated the metabolism of BCAAs in *Clostridium porogenes*. Deletion of porA prevented *Clostridium porogenes* from metabolising BCAAs to produce branched‐chain short‐chain fatty acids (BSCFAs) (Guo et al., 2019) (Figure 1a,b). In another study, it was also confirmed that the gut microbial *Parabacteroides merdae* produced by porA genes is leucine, isoleucine, and valine metabolised to isovalerate (ISV) and 2‐methylbutyrate, respectively, and isobutyrate (ISB) (Ozawa et al., 2005; Qiao et al., 2022) (Figure 1c). This study found that *Parabacteroides merdae* can convert BCAAs into branched‐chain short‐chain fatty acids through the porA gene. Supplementation and directed proliferation can enhance the catabolism of intestinal BCAAs and inhibit activation of the mTOR signalling pathway. These effects ameliorate atherosclerosis in HFD‐induced obese mice, and deletion of porA inhibits branched‐chain amino acid breakdown and improves host metabolic abnormalities in *P. merdae* (Qiao et al., 2022). This suggests the feasibility of gut microbiota‐metabolised BCAAs to provide health benefits and reverse aging.

To evaluate whether the gut microbes are capable of branched‐chain amino acid metabolism and gut microbial metabolites, BCAAs, can be anti‐aging, we passed *Parabacteroides merdae* strain containing porA genes and three metabolites, ISV, 2‐MB, and ISB, which were used to intervene in D‐gal induced aging mice, and the oxidative stress, inflammatory response, and muscle health were evaluated. Combined with the intestinal microbial metagenome and faecal serum nontarget metabolome, the preliminary mechanisms were explored.

### 1.1 Distribution and abundance of porA gene in the human gut microbiomes

To determine the distribution and abundance of the porA gene in human gut microbiomes, we analysed human gut microbiome metagenomics of 13 open cohorts, and a total of 2843 samples. These 13 microbiomes metagenomic cohorts were selected based on three criteria. First, data quality was satisfactory. Second, in addition to healthy people, it also covers a variety of common diseases of the elderly including acute cardiovascular disease (ACVD), colorectal cancer (CRC), Type 2 diabetes (T2D), asthma, and ascites. Third, all cohorts had age tags and included older volunteers. Previous studies have found that the degradation of BCAAs is mediated by the *porA* gene expressed in *Parabacteroides merdae*. It can also provide health benefits to the host (Qiao et al., 2022). When searching for homologous genes in a previous study, the homologous gene BVU_2313 was identified, which is highly similar to the porA gene reported in the literature (Guo et al., 2019). Microbes belonging to the same genera or species, such as Bacteroides, are more likely to share similar patterns in metabolizing BCAAs (Li et al., 2024). In this study, BVU_2313 was used as an index to explore the prevalence of porA in the human gut microbiome.

We quantified the abundance of porA gene using Salmon, an accurate and lightweight tool for quantifying gene abundance. It is widely used in metagenomic gene quantification because of its advantages, such as fast computation speed and small space occupation (Patro et al., 2017). Using the BVU_2313 base sequence (from NCBI) as the reference index, gut microbial metagenomics gene ratio in the sample population were analysed, by integrating the genetic quantitative results, and combining the condition of the samples. All samples were classified according to health status into healthy and diseased populations. To examine the effect of age, the two groups were divided by age, one group for each decade (Figure 1d). In healthy individuals as well as individuals with diseases, porA distributions showed specific differences between different age groups.

In the healthy population, when 51–60 years age group was compared with the 41–50 years group (Figure 1e), the abundance of porA gene significantly increased (*p* < 0.0001); porA gene abundance decreased slightly (*p* < 0.05) when 51–60 years age group was compared with the 61–70 years age group. In the healthy population, the change in abundance of porA gene in the 51–60 years age group was not significant (*p* > 0.05) compared with the 71–80 years and 80 + years age groups.

The results from the diseased population were completely different compared to the healthy population, especially in ACVD and T2D groups, after the age of 70 + years. Compared with 51–60 years of age, the porA gene was significantly reduced (*p* < 0.01) (Figure 1f). The abundance of the porA gene significantly decreased in the elderly stage for populations with ACVD, T2D, and asthma (Figure S1). The porA abundance in the disease group was significantly lower than that in the healthy people group (*p* < 0.05). Therefore, the depletion and deficiency of porA in the elderly may be related to the occurrence and development of various diseases.

### 1.2 Enhancing the metabolism of BCAAs and their BSCFAs can improve oxidative stress in mice

In previous studies, researchers believed that the intestinal microbiota containing the porA gene enhanced the metabolism of BCAAs in the gut, resulting in health benefits. Further exploration of whether metabolites produced by the intestinal *P. merdae* metabolising BCCA can also bring health benefits is essential. Here, we designed an animal study using a D‐galactose induced aging mouse model (Azman & Zakaria, 2019) to explor the effects of gut microbiota that can enhance BCAAs metabolism and their metabolites on aging mice.

BLAST P comparison of BVU_2313 with the existing strain porA gene draft from the School of Food Science, Jiangnan University, was performed to identify strains with the porA gene. Parabacteroides merdae CCFM1428 (identity = 302/360 [83%], positive = 324/360 [90%]), which has high amino acid sequence similarity, was selected for animal experiments. Based on previous literature, we confirm that gut microbes containing the porA gene can metabolize BCAAs to produce three metabolic products, which are 2‐MB (isoleucine metabolite), ISV (leucine metabolite), and ISB (valine metabolite) (Qiao et al., 2022). The amount of BSCFAs in human faecal samples is much lower than that of other short‐chain fatty acids (SCFAs). The total SCFAs content in adult human faeces is approximately 65 mM, while the total BSCFAs content is about 4.5–6.5 mM (Schwarz et al., 2022). Although many animal experiments have verified the role and health benefits of SCFAs, only a few studies have investigated BSCFAs. We explored BSCFAs efficacy in mice; experimental design is shown in Figure 3a, more details please see the experimental methods.

The frailty index (FI) was utilized to represent aging status (Feridooni et al., 2017), with lower scores indicating better health in mice. Among the six experimental groups, mice in the 2‐MB group exhibited the highest aging scores, whereas those in the ISB group had the lowest. The FI analysis revealed that both the gut microbe *P. merdae* and the three BSCFAs were associated with reduced aging scores, suggesting an improvement in the aging condition. (Figure 3b). Liver plays an important role in the metabolism, and histopathological alterations of the liver can reflect many problems associated with aging. We observed histopathological sections of mouse liver and found that D‐galactose induced senescence caused severe focal necrosis (red arrow) and more severe extensive hydrodegeneration (yellow arrow) of mouse hepatocytes (Figure 3c). Gut microbes *P. merdae* reversed part of the liver cell injury, but some stem cell stove necrosis was still observed. ISV, ISB, and 2‐MB groups and the blank group showed similar liver tissue pathological slices. These three types of BSCFAs can alleviate d‐galactose‐induced damage in liver cells.

The progressive accumulation of oxidative damage to macromolecules and mitochondria ultimately leads to pathophysiological changes, functional decline, and accelerated aging. The detrimental consequences of oxidative stress, mainly oxidative damage closely related to accelerated aging and various age‐related diseases, particularly affect lifespan, cardiovascular diseases (CVD), and neurodegenerative diseases (NNDs) (Luo et al., 2020).We examined the antioxidative effect of the gut microbe *P. merdae* and the three BSCFAs in aging mice (Figure 3d–g). The gut microbe *P. merdae* markedly enhanced the antioxidant capacity of aging mice by increasing the activity of GSH‐px and SOD. There were no significant improvements in malondialdehyde (MDA)levels, whereas the gut microbes *P. merdae* improved the CAT enzyme activity in mice.

### 1.3 Enhancing BCAAs metabolism of gut microbiota and its metabolites can improve inflammatory response and confer other health benefits in mice

Chronic inflammation is an important characteristic of aging (Lopez‐Otin et al., 2022). Intestinal ageing is an important factor in this process. There are multiple examples of broad healthspan and lifespanexpanding effects of anti‐inflammatory treatments. Elevated IL‐6 levels in plasma constitute a predictive biomarker of allcause mortality in aging human populations (Hirata et al., 2020). Blockade of TNF‐alpha prevents sarcopenia in mice and improves cognition in aging rats (Sciorati et al., 2020).

**FIGURE 2:**
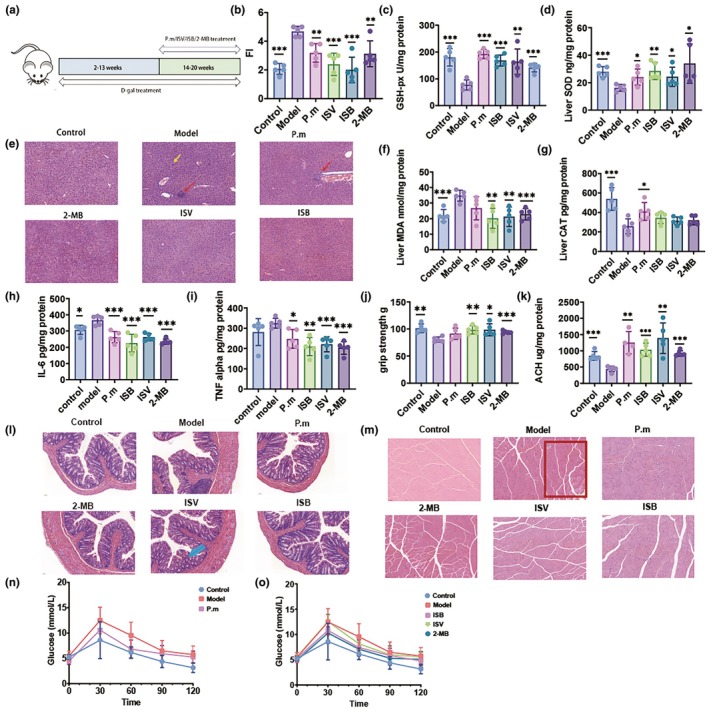
Genetic strain of porA *P. merdae* and three BSCFAs can effectively reverse the frailty index, liver injury, oxidative stress, inflammation, increase the level of brain AchE, improve grip strength and regulate blood glucose level in mice. (a) Diagram of the experimental design. Please see the “Methods” section for additional details. (b) Mouse frailty index. (c) The histopathological alterations of the liver (hematoxylin–eosin (H&E) staining, 20 ×). (d–g) Effect of treatment on GSH‐px, SOD, MDA, and CAT activities in the liver of D‐gal‐induced aging mice in vivo. (d) Changes in liver GSH‐px activity, (e) SOD activity, (f) MDA level (g) CAT activity in the liver. Effects of different treatments on interleukin‐6 (IL‐6) (h) and tumor necrosis factor ɑ (TNF‐ɑ) (i) levels in colonic homogenate. (j) Grip strength of mice. (k) AChE activity in the brain. (l) Histopathological alterations of the colon (hematoxylin–eosin (H&E) staining, 20 ×) (m) Histological morphology of muscle (hematoxylin–eosin (H&E) staining, 20 ×). (n, o) OGTT of mice measured at the end of the intervention. *p < 0.05, **p < 0.01, and ***p < 0.001 indicate significant differences compared with the D‐gal‐treated group. Data shown are mean ± s.e.m. Statistical analysis was performed using T test.

To explore whether enhancing BCAAs metabolism of gut microbiota and its metabolites BSCFAs have different effects on inflammation in aging mice, we measured the levels of interleukin‐6 (IL‐6) (Figure 2h) and tumor necrosis factor ɑ (TNF‐ɑ) (Figure 2i)in colonic homogenate. We found that after the treatment, both IL‐6 and TNF‐alpha levels were reduced in aging mice. The improvement of these inflammatory responses may be related to the following mechanisms. BCAAs boost pro‐inflammatory macrophages and EMT via mTOR signaling (Deng et al., 2024), causing fibrosis. BCAAs also induce insulin resistance by activating mTOR and teaming up with fatty acids. High BCAAs and fatty acid levels stimulate NF‐κB and inflammasomes, leading to inflammation, mitochondrial dysfunction, and metabolic issues (Ye et al., 2020). The treatment regulated BCAAs metabolism in aging mice and therefore improved the inflammatory response. In addition, the histopathological alterations of the colon were observed in aging mice. D‐gal induced aging mice showed colon cell necrosis, fall off the nucleus; treatment with *P. merdae* and three BSCFAs reversed this condition. The morphology and number of intestinal glands in the lamina propria of the colon tissue in the ISV group were altered (Figure 2i). A decline in muscle mass and strength is a common problem in the aging process. We tested the grip strength to check the differences muscle strength in different groups of mice and assessed the histological morphology of the muscle (Seldeen et al., 2018). We found that all treatments, except the *P. merdae* group, were effective in improving the grip strength of the mice (Figure 2j), and the 2‐MB group was the most effective. The muscle fibre spacing of the model group was larger, with loose muscle tissue (red frame), while the other group of muscle fibres were connected closely, with a normal form (Figure 2m). Additionally, we determined the acetylcholinesterase (AChE) activity in the brain (Figure 2k). D‐gal decreased AChE levels in the brain relative to the control group, and all treatments reversed the AChE decrease induced by D‐gal. Finally, BCAAs metabolism has been associated with glycaemic status in many studies. We investigated whether enhancing the metabolism of BCAAs and their metabolites by the gut microbiota could affect the glycaemic status of mice. Glucose tolerance curve analysis (OGTT) was performed. *P. merdae* and the three BSCFAs alleviated the changes in the OGTT induced by D‐galactose (Figure 2n,o), The possible mechanisms are as follows the gut microbiota's BCAAs metabolism influences peripheral serotonin levels and regulates the body's blood sugar health (Li et al., 2024).

### 1.4 Impacts of various treatments on the gut microbiota in aging mice

Imbalance of the gut microbiota, known as dysbiosis, is a significant feature associated with aging. Various research has underscored the relationship between disruptions in the gut's microbial equilibrium and the aging phenomenon. These insights imply that modulating the gut microbiota could potentially enhance both the duration of health and overall life expectancy. To assess how various treatments influence the structure and composition of gut microbiota as mice age. we performed a metagenomic analysis of mouse faeces. Metaphlan 4.0.6 was used for species annotation of the intestinal microbiota and HUMAnN 3.8 was used for functional annotation based on MetaCyc. The gene family files were renamed using the Uniref90 database.

**FIGURE 3:**
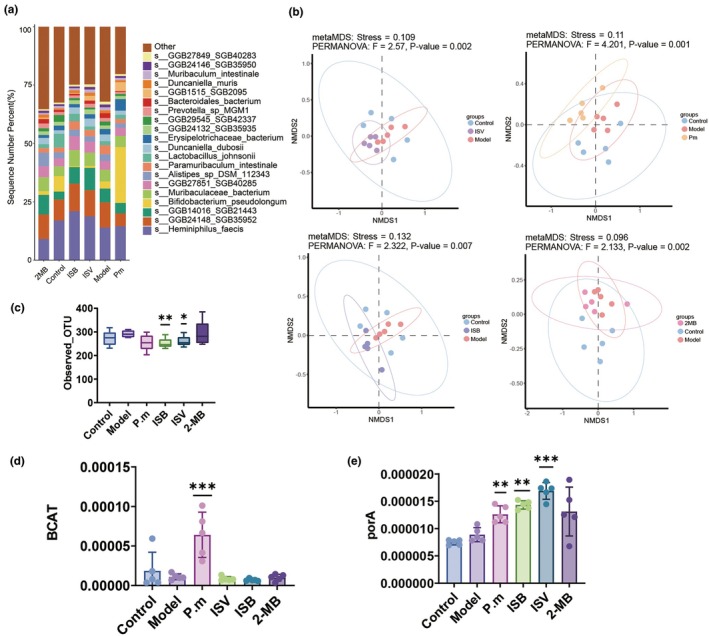
Impacts of various treatments on the gut microbiota in aging mice (a) Gut microbial alterations at the species level. (b) Bata diversity of gut microbiota. (c) Alpha diversity of gut microbiota. (d) Abundance of BCAT genes in the gut microbiota. (e) Abundance of porA genes and their homologs in gut microbes (85% amino acid sequence similarity). *p < 0.05, **p < 0.01, and ***p < 0.001 indicate significant differences compared with the D‐gal‐treated control group. Data shown are mean ± s.e.m. Statistical analysis was performed using T test.

A percentage accumulation map displaying the relative abundances of the top 20 microbial species was generated to highlight changes in gut microbiota. At the species level, *Heminiphilus faecis* was relatively abundant in the ISB, ISV, and control groups. *Bifidobacterium. pseudolongum* and GGB1515_SGB2095 were significantly more abundant in *P. merdae* group than in the other groups. Acetate produced by *Bifidobacterium pseudolongum* has been shown to inhibit hepatocellular carcinoma associated with nonalcoholic fatty liver disease (Song et al., 2023). The abundance of *Muribaculaceae bacterium* increased significantly in the ISV, ISB, and 2‐MB groups (Figure 3a). NMDS, using the Bray‐Curtis index, was employed to examine the beta diversity of gut microbiota across various groups of mice. Compared to the model group, a significant difference was found in *P. merdae*, ISV, and 2‐MB groups (Figure 3b). Alpha diversity was analysed based on the Observed‐OTU index, and the alpha diversity of the intestinal microbes of mice in the ISB and ISV groups decreased slightly. The Alpha diversities of the two groups were similar. According to the accumulation map, the structures in the two groups of mice were similar (Figure 3c).

Based on the gene family files from functional annotation results by HUMAnN 3.8, we analysed changes in the abundance of BCAT enzyme genes and homologous genes of the porA gene in the microbial metabolism of BCAAs which produces BSCFAs mediated by porA. The amino acid sequence of porA gene BVU‐2313 was subjected to BLASTP against the UniRef90 database, identifying gene IDs with over 85% similarity. These IDs were used to query the reannotated gene family files, enabling the identification and quantification of all porA homologous genes. Analysis of the first‐step key metabolic enzyme, BCAT, revealed a significant increase in BCAT abundance in the *P. merdae* group, whereas BCAT abundance in the other groups showed minimal changes (Figure 3d). Significant increases in the abundance of porA were observed in the *P. merdae*, ISB, ISV, and 2‐MB groups (Figure 3e). The mechanism by which intestinal microbiota metabolises BCAAs within the host organism remains unclear. While it has been demonstrated that microbiota harbouring the porA gene can independently metabolise BCAAs, interactions between the gut microbiota may also play a role in regulating the overall BCAAs metabolism in the gut. Second, the ability of gut microbes to metabolise BCAAs, by which gene regulation and metabolism is the key step of speed, is unclear. We hypothesised that in addition to the porA gene, other genes may mediate the ability of the gut microbiota to metabolise BCAAs, although this hypothesis requires further investigation.

**FIGURE 4:**
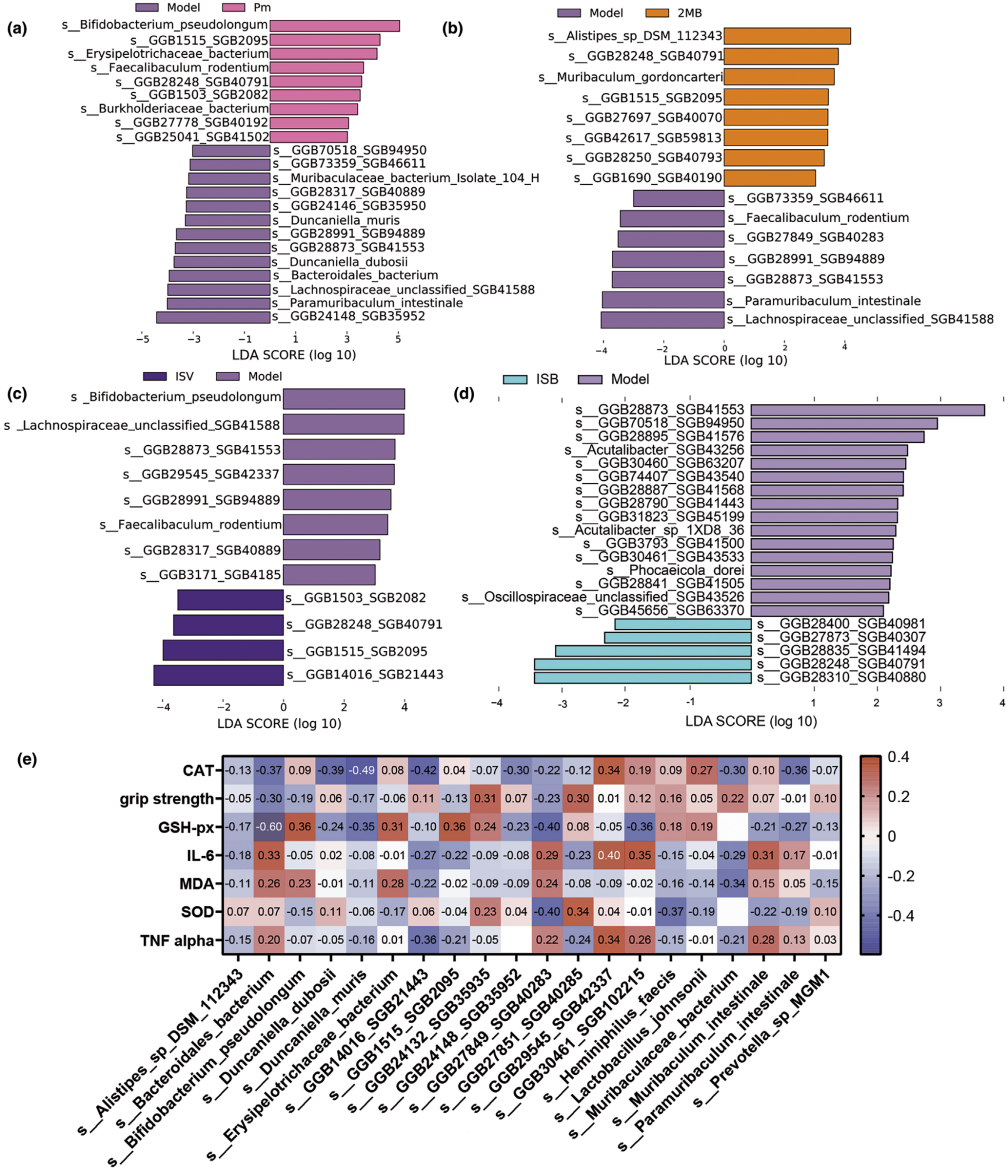
Impacts of various treatments on the gut microbiota in aging mice (a–d) LEfSe analysis between model and the other groups. (e) Spearman correlation analysis between the top 20 species abundance and physiological indicators. *p < 0.05, **p < 0.01, and ***p < 0.001 indicate significant differences compared with the D‐gal‐treated control group. Data shown are mean ± s.e.m. Statistical analysis was performed using T test.

LEFSe analysis was performed to identify key species (LDA3.0) in the gut microbiota of mice in the *P. merdae*, ISB, ISV, 2‐MB, and model groups. The results showed that the abundance of *Bifidobacterium pseudolongum, Erysipelotrichaceae_bacterium, and Faecalibaculum rodentium* increased in *P. merdae* group (Figure 4a). *Erysipelotrichaceae* has been associated with host metabolic disorders and inflammatory diseases (Wu et al., 2021). *Faecalibaculum rodentium* protects against intestinal tumour growth (Zagato et al., 2020). *Faecalibaculum rodentium* modulates duodenal epithelial homeostasis (Cao et al., 2022). *P. merdae* intervention regulates the structure of the gut microbiota in mice to increase the abundance of beneficial bacteria, such as *Bifidobacterium pseudolongum*, alleviating metabolic disorders related to fat metabolism, resulting in health benefits (Bo et al., 2020). The abundance of *Alistipes sp* DSM 112343 was the highest in the 2‐MB group. *Alistipes*, which promotes uric acid excretion (Xu et al., 2024), can metabolise monosaccharide species, reduce blood glucose levels, and improve insulin resistance in obese mice (Takeuchi et al., 2023) (Figure 4b). The abundances of *Bifidobacterium pseudolongum, Faecalibaculum rodentium*, and *Lachnospiraceae unclassified SGB41588* changed in the ISV group. *Lachnospiraceae* primary bile acids can be transformed into secondary bile acids, producing SCFAs and antibacterial material (Abdugheni et al., 2022) (Figure 4c). The abundance of *Oscillospiraceae* was decreased in the ISB group (Figure 4d).

In summary, P. merdae, ISV, and 2‐MB can alter the structure of the mouse gut microbiota. We performed a Spearman correlation analysis between the top 20 species abundance and physiological indicators (Figure 4e). The results showed that the intervention of P. merdae increased the abundance of the beneficial bacterium Bifidobacterium pseudolongum. The Spearman correlation analysis indicated that the increased abundance of *Bifidobacterium pseudolongum* significantly associated with the reduction of inflammatory factors IL‐6 and TNF‐alpha levels.

### 1.5 Impacts of different treatments on the fecal nontargeted metabolome in aging mice

**FIGURE 5:**
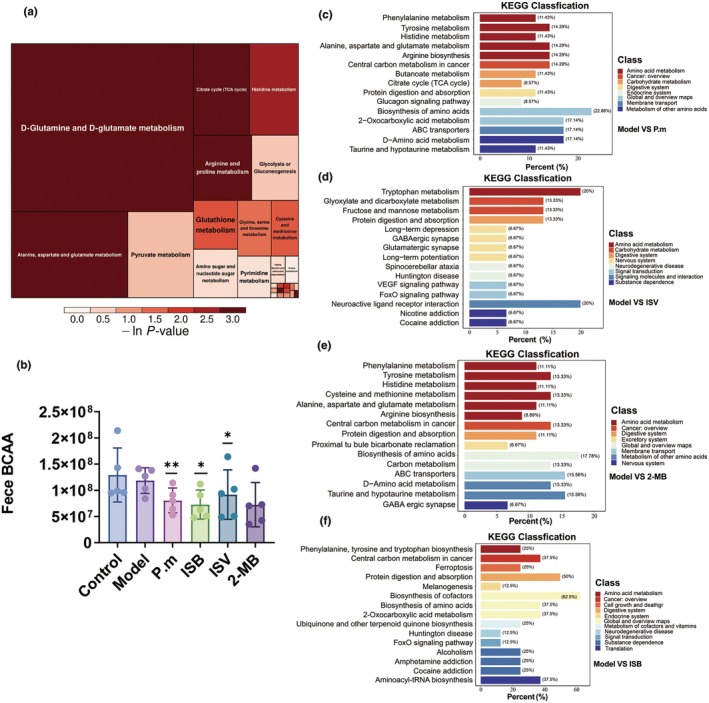
Effects of different treatments on the fecal nontarget metabolome of aging mice. (a) Pathway analysis for group *P.merdae* vs. model (treemap plot). (b) BCAAs content in mice feces (c) KEGG classification for group *P. merdae* vs. model. (d) KEGG classification for group ISV vs. model. (e) KEGG classification for group 2‐MB vs. model. (f) KEGG classification for group ISB vs. model. *p < 0.05, **p < 0.01, and ***p < 0.001 indicate significant differences compared with the D‐gal‐treated control group. Data shown are mean ± s.e.m. Statistical analysis was performed using T test.

To further reveal additional changes in gut microbiota metabolites, nontargeted metabolomics was conducted using mouse faecal samples. Principal component analysis (PCA) was used to analyse differences between metabolites. Significant differences were observed between the *P. merdae*, ISB, ISV, and 2‐MB groups compared to the model group (Figure S2). Volcano plot showed significant difference in metabolites: Compared to the model group, metabolites have significantly increased by 294, and 1446 significantly different metabolites decreased *in the P. merdae group*. Compared with the model group, 526 significantly different metabolites were increased and 334 significantly different metabolites were decreased in the ISB group. Compared to the model group, 1581 significantly different metabolites were increased, and 201 significantly different metabolites were decreased in the ISV group. Compared to the model group, 852 significantly different metabolites were increased, and 938 significantly different metabolites were decreased in the 2‐MB group. Additionally, faecal levels of BCAAs were significantly lower in the *P. merdae* and ISB groups than in the model group (Figure 5a).

KEGG pathway enrichment analysis was performed for differentially expressed metabolites. The abundance of BCAA degradation increased in the *P. merdae* group, which is consistent with previous metagenic results showing an increased abundance of BCAT metabolic enzymes in the *P. merdae* group (Figure 5b). Although previous studies have identified porA as a key gene in the metabolism of BCAAs to produce BSCFAs by *P.merdae*, the key factors that affect the ability of intestinal microbes to metabolise BCAAs remain unknown and deserve further investigation.

Protein digestion and absorption, ABC transporters, arginine biosynthesis, central carbon metabolism in cancer, and amino acid biosynthesis were altered in the *P.merdae* group compared with model group (Figure 5c), After ISV intervention, tryptophan metabolism, protein digestion and absorption, neuroactive ligand‐receptor interaction, and other pathways were significantly altered (Figure 5d). ISV treatment also significantly altered the FoxO and VEGF signalling pathways (Dai & Rabie, 2007), which may partially explain the effectiveness of intestinal microorganisms that metabolise BCAAs to produce ISV in improving obesity‐induced atherosclerotic cardiovascular disease. Unlike ISB and ISV, the 2‐MB group showed altered metabolism of multiple amino acids, including tyrosine, cysteine, and methionine (Figure 5e). ISB interferes with protein digestion, absorption, cofactor biosynthesis, aminoacyl−tRNA biosynthesis, and other pathways. Notably, ISB intervention also altered the FoxO signalling pathway (Lee & Dong, 2017), which may be related to the health benefits of ISB (Figure 5f). FoxO proteins serve as ancient targets for insulin action and are crucial in mediating the effects of insulin on gene expression and metabolism (Unterman, 2018). Genes targeted by FoxO encompass those that code for antioxidant proteins, thereby potentially contributing to the significant role that FoxOs have in the cell's response to oxidative stress (Krafczyk & Klotz, 2022). The health benefits of ISB in terms of glucose health, antioxidant in mice may related to these pathways.

Although all three BSCFAs can alter protein digestion and absorption of the gut microbiota, their health benefits may differ. 2‐MB may alter the synthesis and decomposition of amino acids in the gut microbiota. Metabolic pathway analysis showed that metabolising BCAAs gut microbes and their metabolites, BSCFAs, can affect protein and carbon catabolism of gut microbiota, through a variety of signalling factors to improve overall health.

### 1.6 Impacts of various treatments on the serum metabolome in aging mice

To further explore how the two strains of gut microbes and the three types of BSCFAs affect the overall metabolism of the body, the serum of mice was subjected to nontarget metabolomics. The statistical method of principal component analysis (PCA) was used to analyse differences in metabolites between the groups. *P. merdae*, ISB, ISV, and 2‐MB groups were compared to the model group (Figure S2). Significant differential metabolites are shown as volcano plots. Compared to the model group, *P. merdae* metabolites significantly increased by 948, reducing the metabolic product of a significant difference found in 194. Compared to the model group, the ISB group had 616 significantly increased different metabolites and 308 significantly reduced different metabolites. Compared to the model group, ISV significantly increased 538 metabolites and reduced the metabolic product by a significant difference of 480. Compared to the model group, 410 significantly different metabolites were increased and 317 significantly different metabolites were decreased in the 2‐MB group.

**FIGURE 6:**
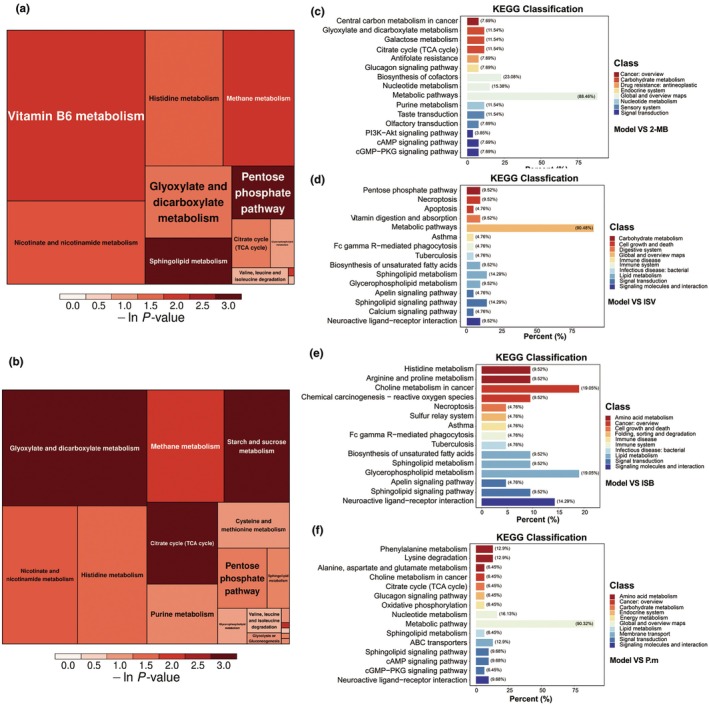
Effects of different treatments on the serum nontarget metabolome of aging mice. (a) Pathway analysis for group ISV vs. Model (treemap plot). (b) Pathway analysis for group 2‐MB vs. Model (treemap plot). (c) KEGG classification for group 2‐MB vs. Model. (d) KEGG classification for group ISV (e) KEGG classification for group ISB vs. Model vs. Model. (f): KEGG classification for group *P. merdae* vs. Model.

KEGG pathway enrichment analysis was performed for differentially expressed metabolites. The ISV group showed a BCAAs degradation pathway with higher abundance (Figure 6a), 2‐MB group mice body of branched chain amino acids degradation also enhance (Figure 6b), and the ISV group for pathway enrichment degree was less than 2‐MB group, suggesting that the combination of aging mice after the intervention of gut flora metabolism changes. We speculate that the ISV group regulates some signalling pathways that affect the metabolism of nutrients, while the 2‐MB group significantly affects the synthesis and metabolism of protein and amino acid nutrients by gut microbiota in aging mice.

Analysis of the serum metabolomics results showed that *P. merdae*, ISV, and 2‐MB significantly altered metabolic pathways. This suggests that the manner in which these treatments work in the body may alter the metabolism. Intervention with 2‐MB altered galactose metabolism, citrate cycle (TCA cycle), and citrate cycle (TCA cycle) cellular signalling pathways (Figure 6c). ISV treatment altered the apelin signalling pathway, Fc gamma R‐mediated phagocytosis, and vitamin digestion and absorption pathways. This suggested that the health benefits conferred by ISV may operate through these pathways (Figure 6d). Intervention in the ISB group altered glycerophospholipid metabolism, biosynthesis of unsaturated fatty acids, and the apelin signalling pathway. This suggests that ISB may exert its effects by regulating the synthesis and metabolism of fatty acid‐related nutrients (Figure 6e). *P. merdae* treatment significantly changed the sphingolipid signalling pathway, which may explain the performance of *P.merdae* in improving metabolic disorders and atherosclerosis (Hla & Dannenberg, 2012). *P.merdae* treatment also altered cAMP signaling (Arumugham & Baldari, 2017), cGMP‐PKG signaling (Hu et al., 2024) and other cell signal transduction‐related pathways, which may explain the observed improvement in glucose metabolism by *P. merdae* (Figure 6f).

In the 2‐MB group, fecal and serum metabolomic results revealed that vitamin B5, regulated by the branched‐chain amino acid valine, is essential for the synthesis of CoA, and cysteine is also a key substance for de novo CoA synthesis. Thus, BCAAs and Cysteine and methionine metabolism can influence the Biosynthesis of cofactors. On the other hand, insulin changes related to the Glucagon signaling pathway can regulate the PI3K − Akt signaling pathway, which strongly stimulates de novo CoA synthesis in response to insulin regulation. Consequently, the impact of valine on CoA synthesis and the stimulation of CoA synthesis by the PI3K − Akt signaling pathway may be associated with blood sugar health (Barritt et al., 2024).

Combining the fecal and serum metabolomic results of the ISB, ISV group, it can be observed that the intervention of ISB has led to changes in amino acid‐related synthetic metabolism in both the serum and intestine of mice. The most striking of these is the change in the FoxO signaling pathway in the gut. The FoxO signaling pathway plays an important role in oxidative stress and regulating cell cycle and apoptosis (Krafczyk & Klotz, 2022). Additionally, the FoxO signaling pathway is a significant factor in age‐related NNDs and T2D (Du & Zheng, 2021).

The fecal and serum metabolomic data from the P.m group indicate changes in phenylalanine metabolism in both the gut and serum of mice, which can suppress the production of TNF‐alpha in pro‐inflammatory macrophages. The alterations in the glucagon signaling pathway are associated with the improvement of glycemic health in mice by P.m (Zhang et al., 2023).

## 2 DISSCUSSION

We investigated whether intervention with *P. merdae*, which has the ability to metabolise BCAAs and produce BSCFAs, could alter the species structure of the gut microbiota in D‐gal‐induced aging mice. The intervention of *P. merdae* and three metabolites BSCFAs can change the species structure of gut microbiota in aging mice, increase the abundance of BCAT and porA genes related to branched‐chain amino acid metabolism, reduce the level of BCAAs in feces, and enhance the metabolism of BCAAs by gut microbiota. This treatment reverses aging by reducing the FI and providing health benefits to oxidative stress, inflammation, and muscle health in aging mice.

In contrast, previous studies have confirmed that *P. merdae* mitigates obesity‐induced atherosclerosis through the metabolism of BCAAs via the porA gene. This study differs from theirs in several key aspects. We extended our investigation to examine the distribution and abundance of the porA gene across various diseases and older age groups, uncovering its potential role in intervening in other diseases and promoting healthy aging. Our exploration of the effects of microbial BCAAs metabolites in the body revealed a positive influence on delayed aging. Gut microbes capable of metabolizing BCAAs and their metabolites can improve oxidative stress, inflammatory responses, frailty indices, acetylcholine levels in the brain, muscle health, and glycemic health in mice, thereby improving physiological conditions and alleviating aging. Preliminary exploration of the mechanisms has shown that the gut microbe Parabacteroides merdae, which can metabolize BCAAs, increases the abundance of beneficial bacteria Bifidobacterium pseudolongum and Faecalibaculum rodentium in the mouse gut, improving the structure of the gut microbiota. The three metabolites produced by gut microbes from BCAAs metabolism, ISV, 2‐MB, and ISB, can alter protein and amino acid metabolism pathways in mice. The intervention with 2‐MB changed the PI3K − Akt signaling pathway in mice, which is related to its performance in glycemic health. The FoxO signaling pathway was altered in the ISV and ISB groups of mice, which may explain the mechanism by which these substances change energy metabolism, improve oxidative stress, promote glycemic health, and thus alleviate aging in mice. Our study represents an initial exploration of the potential of gut microbiota‐mediated BCAAs metabolism to extend healthy aging, offering new insights into the regulation of gut microbiota to promote overall health in aging populations.

The mechanisms by which these three branched‐chain amino acid metabolites confer health benefits remain unclear. In a previous study, ISB and ISV can regulate glucose and lipid metabolism in primary adipocytes, which may help to improve insulin sensitivity in individuals with metabolic disorders (Heimann et al., 2016). ISV has been identified as a ligand for G protein‐coupled receptors that regulate serotonin release in enterochromaffin cells (Bellono et al., 2017).

This study had several limitations that warrant consideration. First, cognitive impairment is prevalent among the elderly population, but our study was constrained by facility resources and regulatory limitations, preventing us from conducting experiments such as Y‐maze and open field tests (Xiao et al., 2021), and limiting our exploration of cognitive studies. Second, this study primarily focused on evaluating the functional role of gut microbiota in metabolising BCAAs and their metabolites to investigate their potential to delay aging. Although we explored the differences and potential mechanisms through intestinal metagenomics and nontargeted metabolomics of serum and stool samples, the specific mechanisms underlying the intestinal microbial metabolism of BCAAs, interactions among gut flora, their mutual regulation with the host, and variations in gut flora structure across different diseases remain to be thoroughly investigated.

Finally, our study focused solely on the porA gene to screen intestinal microorganisms capable of metabolising BCAAs. However, other genes may exist, such as those involved in other metabolic pathways (Agus et al., 2021). Intermediates involved in the gut microbial metabolism of BCAAs also require comprehensive metabolic profiling and the establishment of metabolic gene databases. Further discussion is needed to elucidate the relationship between intestinal microbial metabolism of BCAAs and overall health.

## 3 METHODS

### 3.1 Distribution and abundance of PorA gene in human population

To understand whether the distribution and abundance of porA genes in the human population have specific patterns, we obtained 2814 public human metagenomic datasets (including basic information such as age and sex). The cohort included elderly patients with a variety of common diseases. Literature sources and cohort information are shown in Table S1. Original sequencing reads were analysed using Trimmomatic (version 0.39) to remove sequencing connectors and low‐quality sequences. The filtered sequences were compared to the human reference genome using BWA (version 0.7.17), Samtools (version 1.9), and BEDTools (version 2.30.0) to remove the host sequence. Salmon was used for gene quantification with BVU_2313 as the reference index.

### 3.2 Mice

Animal experiments complied with the current relevant institutional and national animal guidelines and received approval from the Ethics Committee of Jiangnan University, China (IACUC Issue No.: JN.No20230315c0800805[077]). All mice used in this study were 8‐week old male black mice. Specific pathogen‐free C57BL/6J male mice were purchased from Vital River Laboratory Animal Technology Co., Ltd. and housed in the facility at the North District Animal Laboratory of Jiangnan University in Wuxi, China. All mice were provided food and water under 12‐h light/dark cycle in a temperature‐ (22°C ± 1°C) and humidity‐(55% ± 10%) controlled room.

Thirty mice were randomly assigned to different experimental groups (*n* = 5 per group). After entering the feeding facility, the mice were acclimated for 1 week. At 2–13 weeks, an aging model was generated using D‐galactose (CAS No. 59–23‐4, Aladdin).

The control group was subcutaneously injected with sterile saline daily, and the other treatment groups were subcutaneously injected with 700 mg/kg BW/day d‐galactose (dissolved in sterile saline) to ensure that the mice aged to a certain extent before further treatment.

At 14–20 weeks, the treatment was continued with subcutaneous injection of 1000 mg/kg BW/d, because the mice may develop resistance to D‐galactose. Each group of mice was subjected to a corresponding experimental treatment.

The control and model groups were gavaged with 200 μL sterile normal saline daily.

Mice in the *P. merdae* group were treated daily with 200 μL bacterium suspension (live *Parabacteroides merdae* resuspended in sterile saline at a concentration of 2 × 10^8^ c.f.u./mL).

ISV, ISB, and 2‐MB groups were daily gavaged with 6.5 mmol/L, 100 μL/10 g BW isobutyric (ISB), 5.5 mmol/L, 100 μL/10 g BW isovalerate (ISV), and 5.5 mmol/L. 100 μL/10 g BW 2‐methylbutyric acid (2‐MB), respectively. Isobutyric acid, isovaleric acid, and 2‐methylbutyric acid (Aladdin) were dissolved in sterile physiological saline. To evaluate the aging in mice, their FI was evaluated, body weight was measured, fecal samples were collected, and OGTT was performed. The mice were euthanised after these experiments. The liver, brain, small intestine, colon, caecum, and colonic contents were quick‐frozen in liquid nitrogen and transferred to −80°C for storage. Calf muscles, parts of the liver and colon were stored in 4% paraformaldehyde.

### 3.3 Microbial strains

*Parabacteroides merdae* was obtained from the strain bank of Food College of Jiangnan University. Vitamin K (Qingdao Rishui) at a concentration of 1/100 mL and hemin chloride (Qingdao Rishui) were added into BHI medium (Sinopmedicine Shanghai Test) at a concentration of 1/100 mL. The culture medium was placed in an anaerobic workstation for 48 h and the total number of colonies was counted using the plate counting method.

### 3.4 Tissue homogenate preparation and protein quantification

The tissues should be rinsed with ice‐cold PBS (0.01 M, pH = 7.4) to remove excess blood thoroughly. Cut up tissue. About 1.2 g fresh weight of tissue was weighed, and PBS was added at a weight/volume ratio of 1:9. The PBS was added with 1 mL universal protease inhibitors per 100 mL, and the same amount of grinding beads was added to each tissue. Using a precooled tissue grinder mold, the tissue was fully ground on ice until no solids were found. Repeated freeze–thaw tissue homogenates. Centrifuge the homogenate at 5000 × g for 5 ~ 10 min, take the supernatant and separate it for use. BCA method was used to measure the protein concentration. 1.2 mL protein standard preparation solution (0.5 g bovine serum albumin, dissolved in distilled water and constant volume to 100 mL to make 5 mg/mL solution, diluted 10 times over time) was added to one tubulin standard (30 mg BSA), and fully dissolved to make 25 mg/mL protein standard solution. An appropriate amount of 25 mg/mL protein standard was taken and diluted to a final concentration of 0.5 mg/mL. According to the number of samples, 50 volumes of BCA reagent A (weighing 10 g BCA (1%), 20 g Na_2_CO_3_·H_2_O(2%), 1.6 g Na_2_C_4_H_4_O_6_·2H_2_O(0.16%), 4 g NaOH (0.4%), 9.5 g NaHCO_3_(0.95%), respectively), (Add water to 1 L and adjust pH with NaOH or solid NaHCO_3_ to 11.25) add 1 volume of BCA reagent B (take 2 g CuSO4·5H_2_O (4%) and add distilled water to 50 mL) (50:1) to prepare appropriate BCA working solution and mix thoroughly. The standard curve was made by selecting the appropriate concentration within the standard concentration of 0–1.5 mg/mL, and the appropriate dilution was obtained by the sample preexperiment. For formal experiments, 20 μL of samples or different concentrations of standards were added to each well, and 200 μL of LBCA working solution was added when there was no time, and the mixture was incubated at 37°C in the dark for 30 min. Absorbance was measured at 562 nm using a microplate reader. The protein concentration of the samples was calculated.

### 3.5 Tissue biochemical parameters

The levels of MDA and the activities of SOD, CAT, and GSH‐Px in the liver and acetylcholine in the brain were evaluated using assay kits (Shanghai Enzyme‐linked Biotechnology Co., Ltd.) according to the manufacturer's instructions. IL‐6 and TNF‐α in mouse colon tissue were detected by detection kit (R&D) according to the manufacturer's instructions.

### 3.6 Tissue staining

All tissues were stained with HE, and Sevier was commissioned to perform the staining, wax block production, and sectioning.

### 3.7 OGTT

Tail‐pinching adaptation over 5 days reduces the stress response during formal experiments. Mice were fasted overnight for 12 h before the formal experiments, during which the body weight was measured first, and tail tip blood was collected to measure blood glucose. After fasting blood glucose was measured, the mice were given 0.01 mL 20% glucose solution per g body weight by gavage strictly, and the gavage time was recorded. Blood glucose values were measured at 30, 60, 90, and 120 min after gavage and an OGTT curve was drawn.

### 3.8 Mouse FI and grip strength

Before the mice were sacrificed, the FI was determined using the method described by Feridooni et al. modified according to the specific conditions of the mice. The degree of aging was scored for each mouse, which included an assessment of 13 variables. The FI was calculated based on the scores for individual variables as follows: for each variable, a score of 0 represented no disability, 0.5 mild disability, and 1 represented severe disability. The total score was calculated for evaluation and ranged from 0 to 13. See Table S2 for details. The mouse grip strength test was carried out according to the method described by Seldeen et al. (Seldeen et al., 2018) using a grip strength meter (Jinan Yiyan Technology Co., LTD). The mouse was placed flat on the T‐rack, allowing its forepaws to grasp the wire of the grip strength meter. The tail of the mouse was slowly pulled back to read the data; the experiment was repeated five times, and the maximum value was recorded.

### 3.9 Metagenomic sequencing and analysis

Total Stool DNA was extracted using the QIAamp DNA Stool Mini Kit extraction kit, and the purity and integrity of the DNA were detected by 1% agar‐gel electrophoresis. Metagenomic sequencing was performed on the DNBSEQ‐T7 platform. The original sequencing reads used Trimmomatic (version 0.39) to remove sequencing connectors and low‐quality sequences. The filtered sequences were compared with the human reference genome using BWA (version 0.7.17), Samtools (version 1.9) (Li et al., 2009), and BEDTools (version 2.30.0) to remove the host sequence. MetaPhlAn (version 4.0.6) and HUMAnN3 (version 3.8) were used to annotate the species of gut microbiota and metabolic function based on MetaCyc database, respectively, to obtain species‐level abundance information and metabolic pathway abundance information of gut microbiota.

### 3.10 Metabolomics of stool and serum samples

Nontargeted metabolomics detection of mouse serum and fecal samples was performed by Shanghai Baiqu Biomedical Technology Co., LTD. To prepare the samples, 100 μL was mixed with 400 μL of extraction solution (1:1 MeOH:ACN, with deuterated internal standards). This mixture was vortexed for 30 s, sonicated for 10 min at 4°C, and then incubated for 1 h at −40°C to precipitate proteins. After centrifugation at 12,000 rpm (RCF = 13,800 × g) for 15 min at 4°C, the supernatant was transferred to a new glass vial for analysis. Quality control (QC) samples were made by pooling aliquots of the supernatant from all samples. For polar metabolites, analyses were performed using a Vanquish UHPLC system (Thermo Fisher Scientific) with a Waters ACQUITY UPLC BEH Amide column (2.1 mm × 50 mm, 1.7 μm) coupled with an Orbitrap Exploris 120 mass spectrometer (Thermo). The mobile phases were 25 mmol/L ammonium acetate and 25 mmol/L ammonium hydroxide in water (pH 9.75, A) and acetonitrile (B). The auto‐sampler was set to 4°C with a 2 μL injection volume. The Orbitrap Exploris 120 acquired MS/MS spectra in information‐dependent acquisition (IDA) mode using Xcalibur software (Thermo), with ESI source conditions set as follows: sheath gas flow rate at 50 Arb, aux gas flow rate at 15 Arb, capillary temperature at 320°C, full MS resolution at 60,000, MS/MS resolution at 15,000, collision energy at SNCE 20/30/40, and spray voltage at 3.8 kV (positive) or − 3.4 kV (negative).

## AUTHOR CONTRIBUTIONS

*Conceptualization*: H. W., L. F; *Methodology*: L. F., Z. P. and H. W; *Soft‐ware*, L. F; validation, L. F; *Data curation*: L. F., Z. P. and H. W.; Writing—original draft preparation, L. F., and H. W; *Writing—review and editing*, S. L., H. W.; supervision, J. Z., and H. W; *Project administration*: J. Z., S. L., and W. L; and *funding acquisition*: S. L., and W. L. All authors have read and agreed to the published version of the manuscript. Hongchao Wang and Ling Feng contributed equally to this work.

## CONFLICT OF INTEREST STATEMENT

There are no conflicts to declare.

## Supporting information


Table S1.
Table S2.
Table S3.
Figure S1.
Figure S2.
Figure S3.

## Data Availability

The published sequencing data used in this study (Table S3) are available from the Sequence Read Archive (SRA) in the National Center for Biotechnology Information (NCBI) with the accession number PRJNA1180206. For additional information and resource requests, please contact and they will be addressed by the lead contact, Hongchao Wang (hcwang@jiangnan.edu.cn).
