# Supplementary material for: Gut microbiota metabolism of branched‐chain amino acids and their metabolites can improve the physiological function of aging mice
Source: Aging Cell. 2024 Dec 4;24(4):e14434. doi: 10.1111/acel.14434 (PMC11984666; doi:10.1111/acel.14434)
Supplement: Supplementary file 1 — Table S1. Table S2. Table S3. Figure S1. Figure S2. Figure S3. [file ACEL-24-e14434-s001.docx]

Table. S1

|  | study name | PMID | Document source | Other |
| --- | --- | --- | --- | --- |
| 1 | JieZ_2017 | 29018189 | The gut microbiome in atherosclerotic cardiovascular disease | ACVD, healthy |
| 2 | KarlssonFH_2013 | 23719380 | Gut metagenome in European women with normal, impaired and diabetic glucose control | healthy, IGT, T2D |
| 3 | LokmerA_2019 | 30726303 | Use of shotgun metagenomics for the identification of protozoa in the gut microbiota of healthy individuals from worldwide populations with various industrialization levels | healthy |
| 4 | QinJ_2012 | 23023125 | A metagenome-wide association study of gut microbiota in type 2 diabetes | healthy, T2D |
| 5 | QinN_2014 | 25079328 | Alterations of the human gut microbiome in liver cirrhosis | ascites, cirrhosis, healthy, hepatitis, schistosoma, wilson |
| 6 | SchirmerM_2016 | 27984736 | Linking the Human Gut Microbiome to Inflammatory Cytokine Production Capacity | healthy |
| 7 | ThomasAM_2019 | 30936548 | Metagenomic analysis of colorectal cancer datasets identifies cross-cohort microbial diagnostic signatures and a link with choline degradation | CRC, healthy |
| 8 | VogtmannE_2016 | 27171425 | Colorectal Cancer and the Human Gut Microbiome: Reproducibility with Whole-Genome Shotgun Sequencing | CRC, healthy |
| 9 | WampachL_2018 | 30504906 | Birth mode is associated with earliest strain-conferred gut microbiome functions and immunostimulatory potential | healthy |
| 10 | WirbelJ_2018 | 30936547 | Meta-analysis of fecal metagenomes reveals global microbial signatures that are specific for colorectal cancer | CRC, healthy |
| 11 | XieH_2016 | 27818083 | Shotgun Metagenomics of 250 Adult Twins Reveals Genetic and Environmental Impacts on the Gut Microbiome | asthma, generic diabetes, healthy, migraine |
| 12 | YachidaS_2019 | 31171880 | Metagenomic and metabolomic analyses reveal distinct stage-specific phenotypes of the gut microbiota in colorectal cancer | adenoma, carcinoma surgery history, CRC, few polyps, healthy |
| 13 | BritoIL_2016 | 27409808 | Mobile genes in the human microbiome are structured from global to individual scales | healthy |

Table S2. Mouse frailty index

| Body indicators | Potential defects | Body indicators | Potential defects |
| --- | --- | --- | --- |
| Skin | Shedding of hair | Muscle and bone | The gait disorder |
|  | The color of his hair is lighter |  | trembling |
|  | His beard fell |  | Hardening of the tail |
|  | Fur condition |  |  |
| Eye nose status | The corners of the eyes are cloudy | digestion | Rectal prolapse |
|  | Eye nasal secretions |  | The penis prolapse |
|  | Decreased vision |  | diarrhea |

Table S3 accession numbers for each sample

| ccession | sample_name | library_strategy | library_source | library_selection | library_layout | platform | instrument_model | design_description |
| --- | --- | --- | --- | --- | --- | --- | --- | --- |
| SAMN44552678 | C1 | WGS | METAGENOMIC | RANDOM | paired | DNBSEQ | DNBSEQ-T7 | control |
| SAMN44552679 | C2 | WGS | METAGENOMIC | RANDOM | paired | DNBSEQ | DNBSEQ-T7 | control |
| SAMN44552680 | C3 | WGS | METAGENOMIC | RANDOM | paired | DNBSEQ | DNBSEQ-T7 | control |
| SAMN44552681 | C4 | WGS | METAGENOMIC | RANDOM | paired | DNBSEQ | DNBSEQ-T7 | control |
| SAMN44552682 | C5 | WGS | METAGENOMIC | RANDOM | paired | DNBSEQ | DNBSEQ-T7 | control |
| SAMN44552683 | M1 | WGS | METAGENOMIC | RANDOM | paired | DNBSEQ | DNBSEQ-T7 | model |
| SAMN44552684 | M2 | WGS | METAGENOMIC | RANDOM | paired | DNBSEQ | DNBSEQ-T7 | model |
| SAMN44552685 | M3 | WGS | METAGENOMIC | RANDOM | paired | DNBSEQ | DNBSEQ-T7 | model |
| SAMN44552686 | M4 | WGS | METAGENOMIC | RANDOM | paired | DNBSEQ | DNBSEQ-T7 | model |
| SAMN44552687 | M5 | WGS | METAGENOMIC | RANDOM | paired | DNBSEQ | DNBSEQ-T7 | model |
| SAMN44552688 | ISB1 | WGS | METAGENOMIC | RANDOM | paired | DNBSEQ | DNBSEQ-T7 | isb |
| SAMN44552689 | ISB2 | WGS | METAGENOMIC | RANDOM | paired | DNBSEQ | DNBSEQ-T7 | isb |
| SAMN44552690 | ISB3 | WGS | METAGENOMIC | RANDOM | paired | DNBSEQ | DNBSEQ-T7 | isb |
| SAMN44552691 | ISB4 | WGS | METAGENOMIC | RANDOM | paired | DNBSEQ | DNBSEQ-T7 | isb |
| SAMN44552692 | ISB5 | WGS | METAGENOMIC | RANDOM | paired | DNBSEQ | DNBSEQ-T7 | isb |
| SAMN44552693 | ISV1 | WGS | METAGENOMIC | RANDOM | paired | DNBSEQ | DNBSEQ-T7 | isv |
| SAMN44552694 | ISV2 | WGS | METAGENOMIC | RANDOM | paired | DNBSEQ | DNBSEQ-T7 | isv |
| SAMN44552695 | ISV3 | WGS | METAGENOMIC | RANDOM | paired | DNBSEQ | DNBSEQ-T7 | isv |
| SAMN44552696 | ISV4 | WGS | METAGENOMIC | RANDOM | paired | DNBSEQ | DNBSEQ-T7 | isv |
| SAMN44552697 | ISV5 | WGS | METAGENOMIC | RANDOM | paired | DNBSEQ | DNBSEQ-T7 | isv |
| SAMN44552698 | 2MB1 | WGS | METAGENOMIC | RANDOM | paired | DNBSEQ | DNBSEQ-T7 | 2-MB |
| SAMN44552699 | 2MB2 | WGS | METAGENOMIC | RANDOM | paired | DNBSEQ | DNBSEQ-T7 | 2-MB |
| SAMN44552700 | 2MB3 | WGS | METAGENOMIC | RANDOM | paired | DNBSEQ | DNBSEQ-T7 | 2-MB |
| SAMN44552701 | 2MB4 | WGS | METAGENOMIC | RANDOM | paired | DNBSEQ | DNBSEQ-T7 | 2-MB |
| SAMN44552702 | 2MB5 | WGS | METAGENOMIC | RANDOM | paired | DNBSEQ | DNBSEQ-T7 | 2-MB |
| SAMN44552703 | PM1 | WGS | METAGENOMIC | RANDOM | paired | DNBSEQ | DNBSEQ-T7 | P.m |
| SAMN44552704 | PM2 | WGS | METAGENOMIC | RANDOM | paired | DNBSEQ | DNBSEQ-T7 | P.m |
| SAMN44552705 | PM3 | WGS | METAGENOMIC | RANDOM | paired | DNBSEQ | DNBSEQ-T7 | P.m |
| SAMN44552706 | PM4 | WGS | METAGENOMIC | RANDOM | paired | DNBSEQ | DNBSEQ-T7 | P.m |
| SAMN44552707 | PM5 | WGS | METAGENOMIC | RANDOM | paired | DNBSEQ | DNBSEQ-T7 | P.m |

**Figure S1** Distribution and abundance of porA gene in the asthma human gut microbiomes


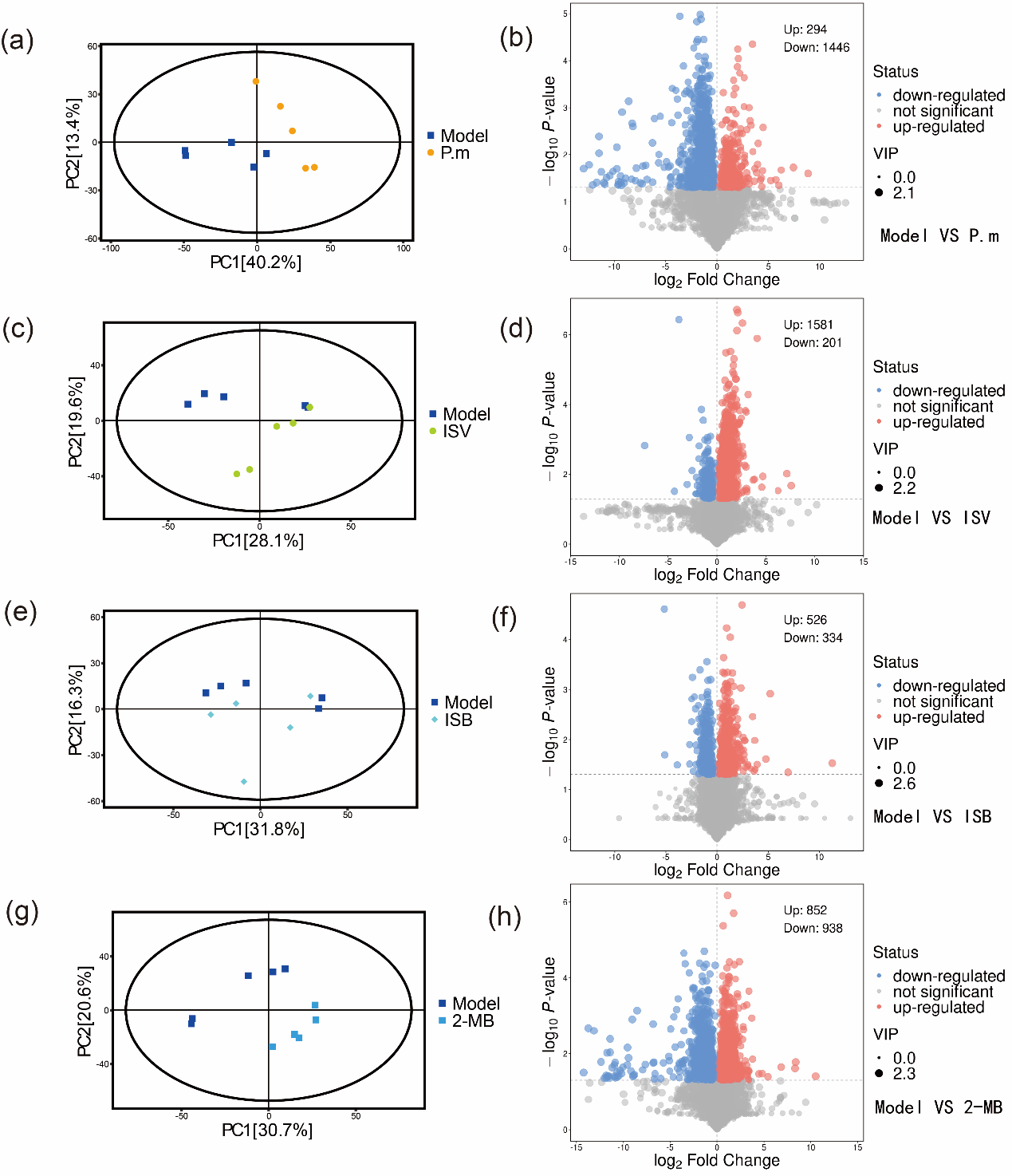


**FIGURE S2**

Effects of different treatments on the fecal non-target metabolome of aging mice. Score scatter plot of PCA model and volcano plot of differential metabolites between each group and model group.


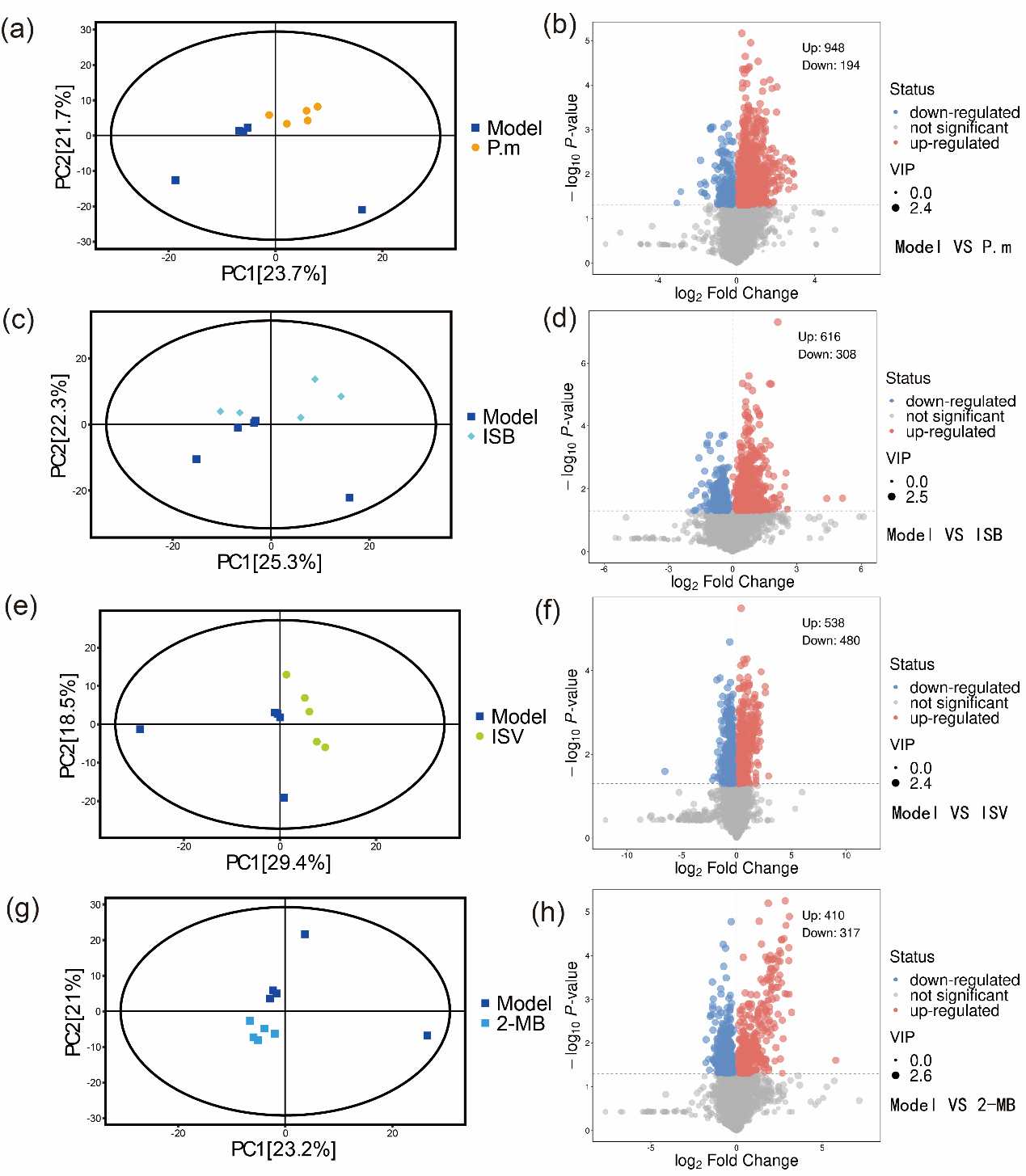


**FIGURE S3**

Effects of different treatments on the serum non-target metabolome of aging mice. Score scatter plot of PCA model and volcano plot of differential metabolites between each group and model group.
